# Time-dependent pharmacokinetic changes and renal SGLT2 expression alterations of dapagliflozin under high-altitude hypoxia in diabetic rats

**DOI:** 10.3389/fphar.2026.1931102

**Published:** 2026-10-26

**Authors:** Yinlian Tong, Chang Wang, Yuemei Sun, Xiaojing Zhang, Huijun Fang, Yuan Xie, Yan Xiao, Shengping Ma, Wenbin Li, Rong Wang

**Affiliations:** 1 Department of Pharmacy, The 940th Hospital of Joint Logistics Support Force of Chinese People’s Liberation Army, Lanzhou, Gansu, China; 2 School of Pharmacy, Ningxia Medical University, Yinchuan, Ningxia, China; 3 Department of Pharmacy, The Second Hospital & Clinical Medical School, Lanzhou University, Lanzhou, Gansu, China; 4 Yushu Tibetan Autonomous Prefecture People’s Hospital, Yushu, Qinghai, China; 5 Ningxia Hui Autonomous Region Fifth People’s Hospital, Shizuishan, Ningxia, China

**Keywords:** dapagliflozin, high-altitude hypoxia, pharmacokinetics, SGLT2, type 2 diabetes mellitus

## Abstract

**Objective:**

This study investigated the time-dependent effects of high-altitude hypoxia on the pharmacokinetics (PK), renal sodium-glucose cotransporter 2 (SGLT2) expression, and glycemic response to dapagliflozin in type 2 diabetes mellitus (T2DM) rats.

**Methods:**

T2DM was induced in male Wistar rats via high-fat diet and low-dose streptozotocin. In the acute study, rats received a single oral dose of dapagliflozin (1 mg/kg) after a 3-day exposure to normoxia or simulated 6000 m hypoxia. In the chronic study, rats received daily dapagliflozin (1 mg/kg) during a 14-day period under normoxia or chronic hypoxia. Plasma dapagliflozin concentrations were determined by UPLC-MS/MS. Fasting blood glucose was monitored serially, and renal cortical SGLT2 protein levels were quantified by ELISA at study endpoint. Individual exposure–response relationships were assessed by Spearman correlation.

**Results:**

Acute hypoxia caused a dramatic PK perturbation: AUC_0-t_ and C_max_ increased by 6.58-fold and 5.06-fold, respectively, compared with acute normoxia (all *p* < 0.01). Chronic hypoxia produced a significant but attenuated effect: AUC_0-t_ and C_max_ increased by 2.11-fold and 1.69-fold, respectively (all *p* < 0.01), representing an approximately 70% reduction in the magnitude of PK change under chronic *versus* acute exposure. Chronic hypoxia was also associated with significantly attenuated early glycemic reduction at days 4 and 7 (*p* < 0.01), though fasting blood glucose levels converged by day 14. Although these observations were temporally associated, a direct causal link is not supported, as SGLT2 was measured only at day 14 whereas glycemic differences emerged earlier (days 4–7). Pooled analysis showed no significant correlation between dapagliflozin exposure and day-14 fasting blood glucose, whereas group-stratified analysis revealed a significant negative correlation between AUC_0-t_ and day-14 fasting blood glucose in normoxic rats only.

**Conclusion:**

Dapagliflozin pharmacokinetics undergo time-dependent adaptation under high-altitude hypoxia. Exposure–response analysis suggested a hypoxia-induced dissociation between dapagliflozin exposure and glycemic response. These findings underscore the need for dedicated clinical PK–PD monitoring in high-altitude populations and provide a descriptive framework for future mechanistic and translational investigations.

## 1 Introduction

The global prevalence of type 2 diabetes mellitus (T2DM) continues to rise, demanding therapeutic strategies that remain effective across diverse physiological and environmental challenges (Sun et al., 2022). Sodium-glucose cotransporter 2 (SGLT2) inhibitors, including dapagliflozin, are a major class of oral antidiabetic drugs (Kasichayanula et al., 2014; Empagliflozin, 2016; Tangri et al., 2024). This drug class lowers blood glucose by inhibiting renal glucose reabsorption.

A significant number of individuals with T2DM reside in or travel to high-altitude regions (>2500 m). These environments are characterized by chronic hypobaric hypoxia, which triggers systemic physiological adaptations (Patil and Singh, 2026; Burtscher et al., 2025) and modulates the expression and activity of drug-metabolizing enzymes and transporters (Luo et al., 2026; Wang et al., 2017; Zhu et al., 2022). Consequently, drug pharmacokinetics (PK) established at sea level may be significantly altered at high altitude (Zhu et al., 2021; Li et al., 2024; Li et al., 2025; Chen et al., 2025). A recent study from the same institution demonstrated that high-altitude exposure significantly altered valproate PK/PD in epileptic rats, with decreased plasma concentrations, reduced AUC, and increased clearance, alongside upregulation of P-gp and hypoxia-inducible factor (HIF)-1α in the blood-brain barrier (Zhang et al., 2026). Similarly, acute high-altitude hypoxia was recently reported to increase the AUC and reduce the clearance of meldonium in rats (Liu et al., 2023). Such PK alterations can lead to unpredictable pharmacodynamic (PD) outcomes.

The impact of hypoxia on drug PK is unlikely to be uniform. Emerging evidence suggests a crucial role for the temporal pattern of exposure (Thiessen et al., 2026; Liao et al., 2025; Zidan et al., 2025; Zhang et al., 2025; Jaśkiewicz et al., 2023; Prieto-Lloret et al., 2021). Acute hypoxia (hours to days) acts as a profound physiological stressor, inducing immediate changes in organ perfusion and rapid modulation of enzyme activity (Thiessen et al., 2026; Liao et al., 2025; Zidan et al., 2025). In contrast, chronic hypoxia (weeks to years) involves acclimatization, with compensatory mechanisms—such as those mediated by HIF—that may modulate the initial acute effects (Zhang et al., 2025; Jaśkiewicz et al., 2023; Prieto-Lloret et al., 2021). For chronic medications like SGLT2 inhibitors, understanding this differential PK/PD landscape is essential for ensuring drug safety during acute altitude exposure and optimizing long-term therapy for high-altitude residents. This rationale forms the basis of our “time-dependent pharmacokinetic modulation” hypothesis.

Despite this clinical relevance, data on SGLT2 inhibitor pharmacokinetics under controlled hypoxic conditions remain scarce (Zhou et al., 2018). This gap is pressing given the intersection of T2DM and chronic hypoxia—a condition involving altered renal hemodynamics and insulin resistance (Woolcott et al., 2015). Existing studies often focus on a single exposure timeframe or assess PK parameters in isolation. A comprehensive, time-resolved investigation integrating PK, drug target expression, and glycemic response is lacking. Moreover, whether hypoxia-induced PK perturbation attenuates with prolonged exposure remains unexplored. We therefore hypothesized that chronic hypoxia would produce a smaller increase in dapagliflozin exposure than acute hypoxia (i.e., fold-change attenuation), accompanied by alterations in renal SGLT2 expression and glycemic response. To test this, we designed parallel acute and chronic exposure studies in T2DM rats.

The dissociation between drug exposure and pharmacological response is a well-recognized phenomenon in clinical pharmacology. Hong et al. demonstrated that carriers of the CYP2C9*3 allele—which encodes a reduced-function drug-metabolizing enzyme—exhibited significantly elevated plasma concentrations of the angiotensin II receptor blocker irbesartan (Hong et al., 2005), yet this pharmacokinetic increase did not translate into enhanced antihypertensive efficacy, likely due to target saturation or physiological compensation. More recently and directly relevant to altitude, Zuo et al. conducted a propensity-matched *post hoc* analysis of the OMAN trial and found that hypertensive patients residing at ∼3000 m on the Tibetan Plateau exhibited significantly attenuated 24-h and daytime blood pressure responses to olmesartan/amlodipine compared with lowland controls (Zuo et al., 2026), despite identical drug regimens and verified medication adherence. These clinical observations raise the question of whether analogous PK-PD dissociation occurs under chronic hypoxia, where both drug disposition and target biology may be concurrently altered.

To address this gap, we conducted a controlled preclinical investigation in T2DM rats simulating both acute and chronic high-altitude hypoxia. The study had three primary objectives: (1) to quantitatively compare PK alterations induced by acute (3-day) *versus* chronic (14-day) hypoxia exposure, providing a direct test of the time-dependency hypothesis; (2) to investigate the effect of sustained chronic hypoxia on renal SGLT2 protein expression and corresponding fasting blood glucose dynamics, enabling an integrated assessment of target-level and systemic responses; and (3) to integrate these PK and PD findings to discuss their implications for the rational use of dapagliflozin in high-altitude populations.

## 2 Materials and methods

### 2.1 Chemical materials and instruments

Dapagliflozin (purity >99%, Lot: D126800) and Canagliflozin (purity ≥99%, Lot: C126191) were obtained from Aladdin Biochemical Technology Co., Ltd. (Shanghai, China) (Figure 1). Streptozotocin (Lot: G2115341) was purchased from Sigma-Aldrich (USA). A high-calorie, high-fat diet (Lot: 2024072501) was provided by Beijing Biotech Co., Ltd. (Beijing, China). Sodium carboxymethyl cellulose (CMC-Na, Lot: 20180412) was purchased from Sinopharm Chemical Reagent Co., Ltd. (Shanghai, China). A Rat SGLT2 ELISA Kit (Lot: 202411) was purchased from Shanghai C-reagent Biotechnology Co. Ltd. Sterile water for injection (Lot: M21041807C) was obtained from Sichuan Kelun Pharmaceutical Co., Ltd. (China). Ammonium acetate (HPLC grade) was sourced from Thermo Fisher Scientific (Waltham, MA, USA). Methanol and acetonitrile (HPLC grade) were purchased from Merck Company, Inc.

**FIGURE 1:**
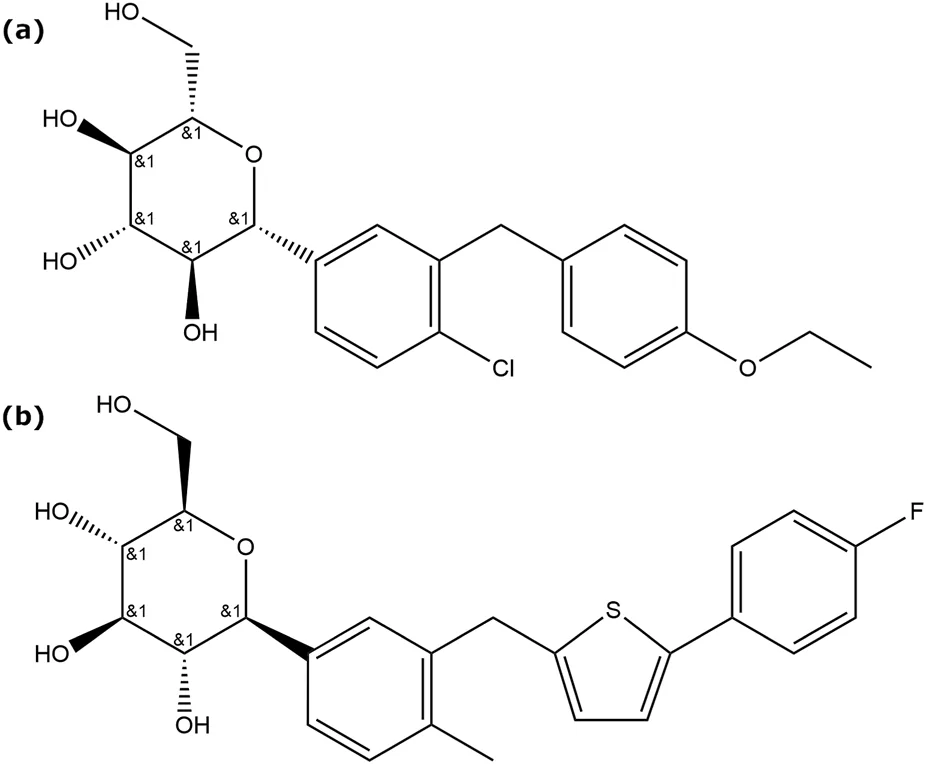
Molecular structure diagrams of dapagliflozin **(a)** and canagliflozin **(b)**.

All analyses utilized an ultra-performance liquid chromatography–tandem mass spectrometry (UPLC–MS/MS) system. This system consisted of a Thermo Scientific™ UltiMate™ 3000 series UPLC coupled with a TSQ Quantum Access™ triple quadrupole mass spectrometer. The mass spectrometer featured an electrospray ionization (ESI) source. Additional instruments included an electronic analytical balance (Mettler-Toledo, USA), a high-speed refrigerated centrifuge (Shanghai Anting Scientific Instrument Factory, China), a blood glucose monitoring system (Accu-Chek Performa, Roche, Germany), and a custom-designed hypobaric hypoxia chamber (Guizhou Fenglei, China). The hypobaric chamber simulated altitudes ranging from 0 to 8000 m.

### 2.2 Animals and ethical approval

Specific pathogen-free (SPF) male Wistar rats (initial weight 180–220 g) were supplied by Beijing Huafukang Bio-technology Co., Ltd. (license No. SCXK (Beijing) 2024–0003). Animals were acclimatized for 1 week under standard laboratory conditions: temperature 23 °C ± 2 °C, relative humidity 50% ± 10%, and a 12 h/12 h light/dark cycle, with free access to standard rodent chow and water. All procedures strictly followed the National Institutes of Health Guide for the Care and Use of Laboratory Animals and were approved by the Ethics Committee on Animal Experimentation of the 940th Hospital of Joint Logistic Support Force (Approval No. 2024KYLL326D). The researchers implemented all possible measures to minimize animal suffering. These measures included the use of isoflurane anesthesia during invasive procedures and the strict application of predefined humane endpoints.

### 2.3 Induction of the T2DM rat model

The study employed a well-characterized protocol to establish the T2DM model (Reed et al., 2000; Wan et al., 2025). This protocol combined dietary manipulation with low-dose chemical induction. Specifically, the rats received a high-calorie, high-sugar diet for 4 consecutive weeks. This dietary regimen aimed to induce insulin resistance. On day 29, following a 12-h fast, each rat received a single intraperitoneal injection of freshly prepared streptozotocin (30 mg/kg). The research team dissolved the streptozotocin in 0.1 mol/L citrate buffer (pH 4.5). Fasting blood glucose levels were measured from the tail vein 72 h post-injection. A glucometer (Accu-Chek Performa, Roche) performed these measurements. The study considered rats with fasting blood glucose levels persistently ≥11.1 mmol/L on three consecutive measurements as successfully diabetic. Only these successfully induced diabetic rats proceeded to the subsequent pharmacokinetic studies.

### 2.4 Study design and hypoxic exposure

To test the time-dependency hypothesis, we established two distinct exposure protocols: a 3-day acute exposure protocol designed to reflect the physiological stress of rapid ascent, and a 14-day chronic protocol to simulate sustained high-altitude residence with potential acclimatization. The acute study assessed single-dose PK under an established hypoxic state, while the chronic study evaluated steady-state PK, serial fasting blood glucose dynamics, and renal target expression at the endpoint. In both the acute and chronic pharmacokinetic studies, rats were fasted overnight for 12 h with free access to water before dapagliflozin administration.

#### 2.4.1 Acute hypobaric hypoxia exposure and pharmacokinetic assessment

The researchers randomly assigned T2DM rats to two groups: Acute Normoxia (A-N, n = 8) and Acute Hypoxia (A-H, n = 8). The A-H group spent a 3-day acclimatization period inside a hypobaric chamber set to simulate 6000 m altitude. Subsequently, while remaining under hypoxic conditions, this group received a single oral dose of dapagliflozin (1 mg/kg). The drug was suspended in 0.5% CMC-Na. The A-N group underwent an identical 3-day period under normoxic conditions followed by the same dosing procedure. Blood samples (∼0.15 mL) were collected from the retro-orbital plexus under light isoflurane anesthesia (3% induction, 1.5% maintenance, with room air as carrier gas). Sampling time points were pre-dose (0 h) and at 0.08, 0.17, 0.33, 0.5, 0.75, 1, 1.5, 2, 4, 6, 8, 12, and 24 h post-dose. This design characterized the single-dose pharmacokinetics under an established state of acute hypoxic stress.

#### 2.4.2 Chronic hypobaric hypoxia exposure and pharmacokinetic assessment

A separate cohort of T2DM rats was randomly divided into Chronic Normoxia (C-N, n = 8) and Chronic Hypoxia (C-H, n = 8) groups. The C-H group resided continuously at a simulated 6000 m altitude for 14 days (Wan et al., 2025). This group also received once-daily oral administrations of dapagliflozin (1 mg/kg/day) throughout this period (Sato et al., 2022; Qiuxiao et al., 2024).

This chronic study had three primary components. First, the study assessed steady-state pharmacokinetics. On day 14, an intensive pharmacokinetic sampling session was conducted immediately after the final dose. The sampling schedule mirrored that of the acute study. This characterized pharmacokinetics under chronic hypoxic adaptation. Second, the study involved serial monitoring of fasting blood glucose. Measurements were taken at baseline (day 0) and subsequently on days 1, 4, 7, 10, and 14. Meanwhile, approximately 1–2 μL of blood was collected from the tail vein of rat after a 12-h fast, followed by immediate analysis using a portable glucose meter (Accu-Chek Performa, Roche). A single investigator conducted all measurements to ensure consistency. Third, the study evaluated renal target expression at the experimental endpoint. On day 14, after the final pharmacokinetic blood sample, rats were deeply anesthetized with isoflurane (5% induction, 3% maintenance) and euthanized by rapid cervical dislocation by trained personnel (AVMA guidelines), avoiding chemical interference with PK/SGLT2 assays. Renal cortices were then collected. A 10% (w/v) tissue homogenate was prepared in PBS buffer (pH 7.4). The SGLT2 protein levels in the homogenate supernatant were quantified using a commercial ELISA Kit. The assay followed the manufacturer’s instructions, with a linear range of 12 ng/L to 400 ng/L.

### 2.5 Drug administration and sample collection

The team collected all blood samples into pre-chilled, heparinized tubes. Centrifugation of the samples occurred within 30 min of collection (4,000 × g, 10 min, 4 °C). The resulting plasma was aliquoted, rapidly frozen in liquid nitrogen, and subsequently stored at −80 °C. Storage continued until UPLC-MS/MS analysis. This standardized protocol was applied rigorously to all samples from both the acute and chronic studies. Strict adherence ensured sample integrity and minimized pre-analytical variability.

### 2.6 Bioanalytical method: UPLC-MS/MS quantification

Chromatography employed a Hypersil GOLD™ C18 column (2.1 × 100 mm, 3.0 μm) at 30 °C. Mobile phase: (A) 20 mM ammonium acetate in water (B) acetonitrile: 5 mM ammonium acetate (9:1, v/v). Gradient: 0–0.9 min, 15%→85% B; 0.9–1.7 min, 85% B; 1.7–2.2 min, 85%→15% B; 2.2–3.5 min, 15% B. Flow rate: 0.35 mL/min. The autosampler temperature was set at 4 °C and the injection volume was 10 μL.

The mass spectrometer operated in negative electrospray ionization (ESI) mode with selected reaction monitoring (SRM). The ion source temperature and spray voltage were set to 270 °C and 3.0 kV, respectively. The vaporizer temperature was set to 40 °C. Nitrogen served as the sheath and auxiliary gas at pressures of 35 Arb and 10 Arb, respectively. Analyses monitored a stable acetate adduct ([M + CH3COO]-). The specific SRM transitions were *m/z* 467.3→329.2 (CE 19 eV; Tube Lens 42 V) for dapagliflozin and *m/z* 503.4→365.2 (CE 21 eV; Tube Lens 48 V) for canagliflozin (internal standard, IS).

Calibration standards (10–1000 μg/L) and quality control (QC) samples (30, 200, 800 μg/L) were prepared in blank rat plasma. For sample processing, 50 μL of plasma was mixed with 100 μL of acetonitrile. This acetonitrile contained the IS at a concentration of 250 μg/L. After vortexing and centrifugation, the supernatant was injected for analysis.

The bioanalytical method was fully validated according to ICH M10 guidelines. The validation process assessed selectivity, linearity, the lower limit of quantification (LLOQ, 10 μg/L), accuracy, precision, matrix effects, recovery, and stability. All tested parameters met the pre-defined acceptance criteria.

### 2.7 Pharmacokinetic analysis

Non-compartmental pharmacokinetic analysis was performed on the individual plasma concentration-time profiles. DAS software (version 3.2.8) facilitated this analysis. The estimated parameters included the area under the plasma concentration-time curve from time zero to the last measurable concentration (AUC_0-t_) and extrapolated to infinity (AUC_0-_ ∞ ), the maximum plasma concentration (C_max_) and the time to reach C_max_ (T_max_), the terminal elimination half-life (t_1/2z_), the apparent volume of distribution during the terminal phase (Vz/F), the apparent oral clearance (CLz/F), and the mean residence time (MRT).

### 2.8 Statistical analysis

All data are expressed as mean ± standard deviation (SD). Sample size (n = 8 per group) was chosen based on previous pharmacokinetic studies in rat models, which typically have 80% power to detect a 1.5-fold difference in AUC at α = 0.05; however, no formal power analysis was performed for this exploratory study, a limitation we acknowledge.

For pharmacokinetic parameters, a two-way analysis of variance (ANOVA) was used to assess the main effects of “Hypoxic Treatment” (Normoxia vs. Hypoxia) and “Exposure Duration” (Acute vs. Chronic), as well as their interaction. When a significant interaction (*p* < 0.05) was detected, planned comparisons were conducted using independent samples *t*-tests: (1) A-H vs. A-N; (2) C-H vs. C-N; and (3) A-H vs. C-H. For chronic study data (fasting blood glucose at each time point and renal SGLT2 at study terminus), independent samples *t*-tests were similarly used to compare the C-H and C-N groups. For the individual-level exposure–response analysis, Spearman’s rank correlation was used to assess the relationship between dapagliflozin exposure metrics (AUC_0-t_ and C_max_) and fasting blood glucose at day 14. Group-stratified Spearman correlations and linear models including group as a covariate were also performed. Pharmacokinetic and glycemic comparisons were performed using IBM SPSS Statistics (version 22.0); exposure–response analyses and figure generation were performed using R software (version 4.6.1). In all analyses, *p* < 0.05 was considered statistically significant. When homogeneity of variance was violated (Levene’s test), robustness was verified by non-parametric alternatives or interpreted alongside effect sizes.

For serial fasting blood glucose measurements, *t*-tests at each time point were planned exploratory comparisons. The same exploratory approach was applied to the exposure–response correlations using day-4 and day-7 fasting blood glucose. Given the hypothesis-generating nature of these endpoints and the limited number of pre-specified time points (days 0, 1, 4, 7, 10, 14), no adjustment for multiple comparisons (e.g., Bonferroni) was applied. Nevertheless, the observed differences at days 4 and 7 remained significant after Bonferroni correction (adjusted α = 0.0083).

## 3 Results

### 3.1 Bioanalytical method validation

#### 3.1.1 Selectivity, specificity, and carryover

Selectivity assessments confirmed an absence of significant endogenous interference at the retention times of dapagliflozin and the internal standard. Their respective retention times were 2.01 min and 2.12 min. Carryover was negligible. Representative chromatograms are shown in Figure 2.

**FIGURE 2:**
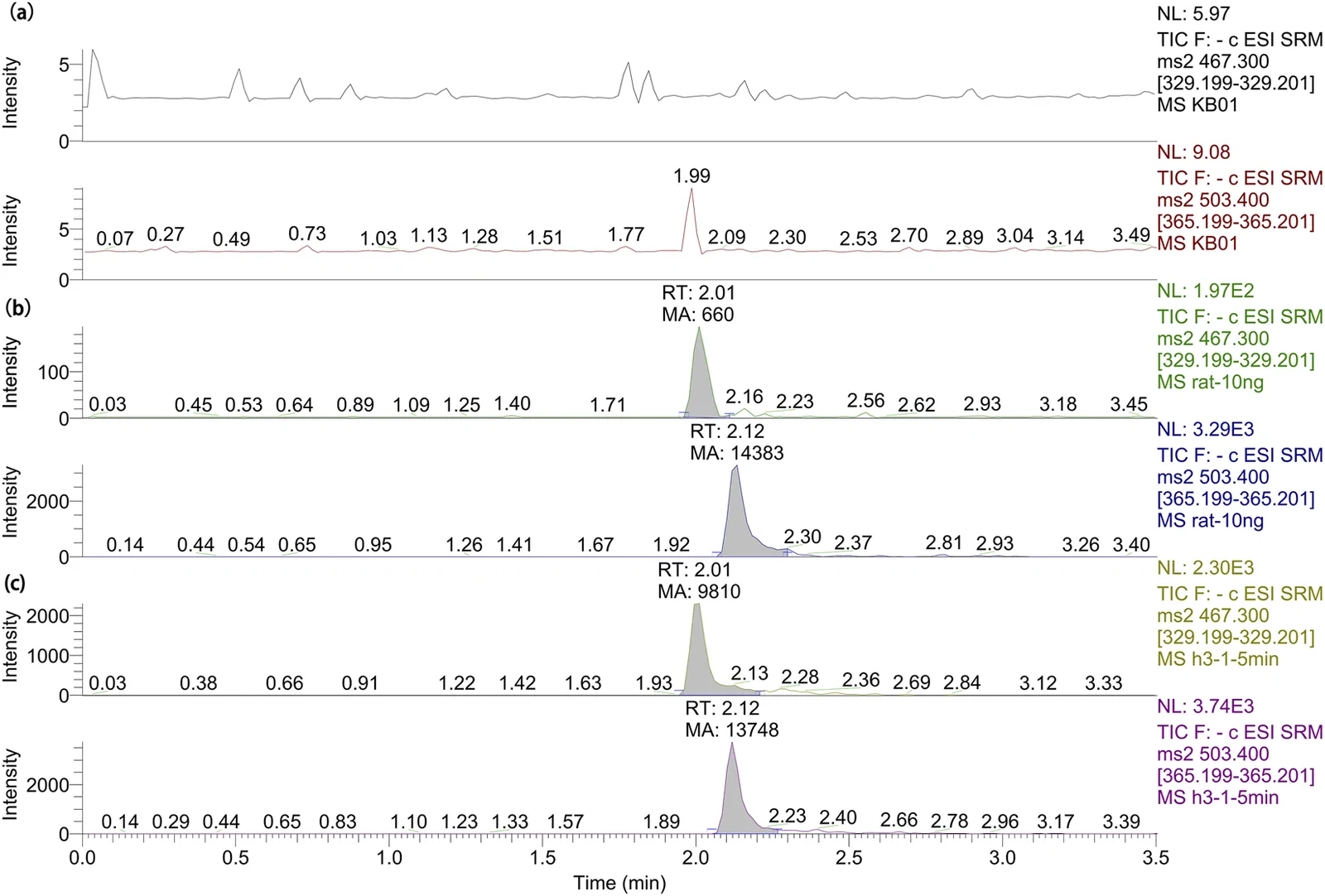
Representative chromatograms for dapagliflozin and IS. Note: **(a)** blank rat plasma; **(b)** blank rat plasma containing LLOQ concentration; **(c)** Rat plasma sample collected 5 min after a single dose of dapagliflozin.

#### 3.1.2 Linearity and LLOQ

The method demonstrated excellent linearityacross the calibration range of 10–1000 μg/L. Weighted (1/x^2^) linear regression yielded correlation coefficients (R^2^) ≥ 0.9935 in three independent runs (Table 1). The lower limit of quantification (LLOQ) was validated at 10 μg/L. Accuracy and precision at the LLOQ were acceptable.

**TABLE 1:** Linear ranges, correlation coefficients of dapagliflozin.

Linear range (μg/L)	Regression equation	R^2^
10.0–1000.0	Y = −0.0133509 + 0.0045139*X	0.9968
	Y = 0.020146 + 0.00460182*X	0.9944
	Y = 0.047287 + 0.0044117*X	0.9935

#### 3.1.3 Precision and accuracy

Intra-day and inter-day precision (coefficient of variation, CV) for dapagliflozin at all QC levels were within 11% and 10%, respectively (Table 2). Accuracy ranged from 94.50% to 103.18% intra-day and from 93.90% to 100.91% inter-day, meeting the acceptance criteria (±15% for QCs).

**TABLE 2:** Intra-day and Inter-day precision and accuracy of dapagliflozin.

QC (μg/L)	Intra-day (n = 6)		​	Inter-day (n = 18)		​
	Measured value(μg/L)	CV (%)	Accuracy (%)	Measured value(μg/L)	CV (%)	Accuracy (%)
10.0	9.45 ± 1.02	10.79	94.50	9.39 ± 0.85	9.05	93.90
30.0	28.38 ± 2.81	9.90	94.60	29.30 ± 2.06	7.03	97.67
200.0	200.13 ± 16.77	8.38	100.07	196.38 ± 12.23	6.23	98.19
800.0	825.42 ± 40.72	4.93	103.18	807.29 ± 39.99	4.95	100.91

#### 3.1.4 Matrix effects and extraction recovery

Matrix effects were minimal, with values ranging from 84.88% to 89.71%. The extraction recovery of dapagliflozin was consistent and efficient, averaging between 80.25% and 82.94% across low, medium, and high QC levels (Table 3).

**TABLE 3:** Matrix effect and extraction recovery of dapagliflozin (n = 6).

QC (μg/L)	Matrix effect(%)	Extraction recovery (%)
	Value	Value
30.0	89.71 ± 3.02	82.94 ± 5.92
200.0	84.88 ± 3.14	80.25 ± 3.87
800.0	86.83 ± 6.61	80.53 ± 2.83

#### 3.1.5 Stability

Dapagliflozin was stable in rat plasma under various storage conditions. After 24 h at room temperature, deviations from nominal concentrations were within ±7.71% (Table 4). Stability was also confirmed after three freeze-thaw cycles (within ±6.14%), and during long-term storage at −80 °C for 14 days (within ±5.52%).

**TABLE 4:** Sample storage stability studies of dapagliflozin (%, n = 6).

QC (μg/L)	RT 25 °C (24 h)	AS 4 °C (24 h)	4 °C (48 h)	RFT (48 h)	−80 °C (14d)
30.0	−7.71	−6.30	−4.51	−6.14	−4.99
200.0	−5.02	−3.91	−6.52	−4.35	−5.52
800.0	3.60	3.63	0.76	−1.30	2.65

RT, room temperature; AS, autosampler; RFT, repeated freezing and thawing thrice.

#### 3.1.6 Dilution effects

The 10-fold dilution of a sample with a concentration exceeding the ULOQ resulted in an accuracy between 93.42% and 104.90% and a precision between 3.41% and 6.22%, confirming the integrity of the dilution procedure.

### 3.2 Pharmacokinetic profiles under acute and chronic hypoxic exposure

The mean plasma concentration-time profiles of dapagliflozin are shown in Figure 3. Plasma concentrations were markedly elevated in both hypoxic groups compared with normoxic controls, with a more pronounced increase after acute exposure. Early-phase profiles (0–4 h) revealed that concentrations in the A-H group exceeded those in the A-N group as early as 0.5 h post-dose. This rapid elevation, together with the significantly reduced Vz/F and CLz/F observed in the A-H group (Table 5), suggests that altered apparent volume of distribution and/or apparent oral clearance—rather than enhanced absorption alone—may contribute to the early rise. However, because dapagliflozin was administered orally, these apparent parameters cannot distinguish changes in oral bioavailability from true changes in distribution or elimination; therefore, a contribution from altered first-pass metabolism or oral bioavailability cannot be excluded.

**FIGURE 3:**
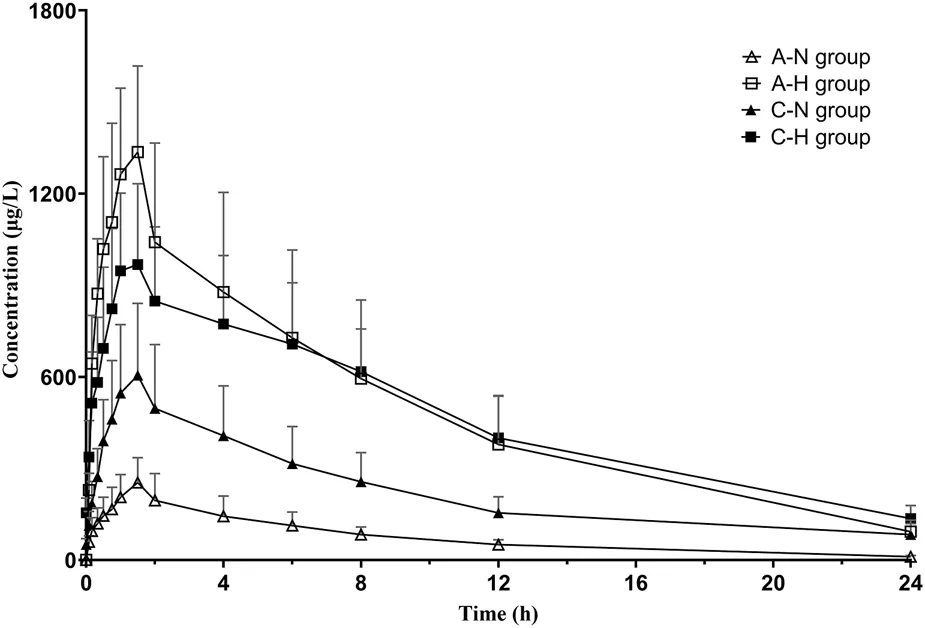
Mean plasma concentration-time profiles of dapagliflozin in T2DM rats following a single oral dose (1 mg/kg) under acute (3-day) or chronic (14-day) normoxic or hypoxic conditions. A-N, Acute Normoxia (n = 8); A-H, Acute Hypoxia (n = 8); C-N, Chronic Normoxia (n = 8); C-H, Chronic Hypoxia (n = 8). Data are presented as mean ± SD.

**TABLE 5:** Main pharmacokinetic parameters of dapagliflozin in T2DM rats under normoxic, acute hypoxic, and chronic hypoxic conditions (mean ± SD, n = 8).

Parameter (Unit)	A-N group	A-H group	C-N group	C-H group	Statistical outcome (two-way ANOVA)	Interaction (*p*-value)	Levene’s test (*p*-value)
AUC_0-t_ (μg·L^-1^·h)	1785.38 ± 613.31^a^	11753.85 ± 4214.72^b^	5351.01 ± 1919.39^a^	11267.03 ± 2904.71^c^	**H: *p* < 0.001, η** ^**2**^ **= 0.704** D: *p* = 0.125	**0.046**	0.065
AUC_0-_ ∞ (μg·L^-1^·h)	1865.63 ± 646.88^a^	12558.11 ± 4512.58^b^	6171.83 ± 1729.85^a^	12758.95 ± 3404.02^c^	**H: *p* < 0.001, η** ^**2**^ **= 0.707** **D: *p* = 0.041, η** ^**2**^ **= 0.141**	0.061	**0.034**
C_max_ (μg·L^-1^)	264.32 ± 87.27^a^	1336.31 ± 281.89^b^	605.25 ± 235.38^a^	1023.27 ± 248.52^c^	**H: *p* < 0.001, η** ^**2**^ **= 0.756** D: *p* = 0.863	**< 0.001**	0.076
T_max_ (h)	1.50 ± 0.27	1.50 ± 0.00	1.5 ± 0.00	1.31 ± 0.26	H: *p* = 0.165 D: *p* = 0.165	0.165	**0.001**
t_1/2z_ (h)	5.26 ± 0.39	5.99 ± 0.32	8.57 ± 2.71	7.43 ± 1.03	H: *p* = 0.701 **D: *p* < 0.001, η** ^**2**^ **= 0.427**	0.082	**<0.001**
Vz/F (L·kg^-1^)	4.48 ± 1.54^a^	0.74 ± 0.17^b^	2.19 ± 1.00^a^	0.89 ± 0.23^c^	**H: *p* < 0.001, η** ^**2**^ **= 0.679** **D: *p* = 0.003, η** ^**2**^ **= 0.278**	**0.001**	**0.001**
CLz/F (L·h^-1^·kg^-1^)	0.59 ± 0.20^a^	0.09 ± 0.02^b^	0.17 ± 0.04^a^	0.08 ± 0.02^c^	**H: *p* < 0.001, η** ^**2**^ **= 0.703** **D: *p* < 0.001, η** ^**2**^ **= 0.546**	**< 0.001**	**0.002**
MRT_0-t_ (h)	6.62 ± 0.48	7.10 ± 0.37	7.88 ± 0.72	8.05 ± 0.63	H: *p* = 0.115 **D: *p* < 0.001, η** ^**2**^ **= 0.520**	0.440	0.635
MRT_0-_ ∞ (h)	7.68 ± 0.65	8.73 ± 0.54	12.23 ± 3.32	11.13 ± 1.52	H: *p* = 0.968 **D: *p* < 0.001, η** ^**2**^ **= 0.496**	0.115	**<0.001**

H: hypoxia main effect; D: Duration main effect. For significant main effects (*p* < 0.05), partial eta-squared (η^2^) is reported. Interaction *p*-values in bold indicate statistical significance (*p* < 0.05). Different superscript letters (^a,b,c^) within a row indicate statistically significant differences (*p* < 0.05) based on independent samples t-tests (planned comparisons). Levene’s test p-values <0.05 (in bold) indicate violation of the homogeneity of variance assumption.

Key PK parameters are summarized in Table 5. Two-way ANOVA revealed highly significant main effects of Hypoxic Treatment on AUC_0-t_, AUC_0-_ ∞ , C_max_, Vz/F, and CLz/F (all *p* < 0.001, large effect sizes η^2^ > 0.67), confirming that hypoxia profoundly altered dapagliflozin disposition. Significant main effects of Exposure Duration were also observed for t_1/2z_, Vz/F, CLz/F, MRT_0-t_, MRT_0-_ ∞ , and AUC_0-_ ∞ (*p* < 0.05 for all). Most critically, significant interactions between Hypoxic Treatment and Exposure Duration were identified for AUC_0-t_ (*p* = 0.046), Vz/F (*p* = 0.001), and CLz/F (*p* < 0.001), validating that the hypoxic effect on systemic exposure and clearance was duration-dependent.

Given these significant interactions, planned comparisons were conducted to quantify the effect of hypoxia within each exposure duration. Compared with the Acute Normoxia group, acute hypoxia increased AUC_0-t_ and C_max_ by 6.58-fold and 5.06-fold, respectively, while decreasing Vz/F and CLz/F by 83.5% and 84.7% (all *p* < 0.01). In contrast, the effect of chronic hypoxia was markedly attenuated: compared with the Chronic Normoxia group, chronic hypoxia increased AUC_0-t_ and C_max_ by 2.11-fold and 1.69-fold, respectively, with corresponding decreases of 59.4% and 52.9% in Vz/F and CLz/F (all *p* < 0.01). Expressed as fold-change relative to each group’s normoxic baseline, the hypoxia-induced increase in systemic exposure was reduced by approximately 70% under chronic *versus* acute exposure—a quantitative evidence of time-dependent PK adaptation. Although absolute AUC_0-t_ values did not differ significantly between the two hypoxic groups (*p* = 0.792), this does not negate the adaptive pattern; the attenuation is appropriately measured by the fold-change reduction relative to duration-matched normoxic controls, rather than by direct comparison of absolute values between A-H and C-H. Similarly, Vz/F and CLz/F showed no significant differences between A-H and C-H (*p* = 0.169 and 0.869, respectively).

Of note, the interaction term reached significance for AUC_0-t_ but not for AUC_0-_ ∞ (*p* = 0.061), likely reflecting greater inter-individual variability in the extrapolated terminal phase (Levene’s test for t_1/2z_, *p* < 0.001). Accordingly, our primary interpretation of time-dependent attenuation relies on AUC_0-t_ and C_max_, which are less influenced by extrapolation.

### 3.3 Effect of chronic hypoxia on fasting blood glucose

Fasting blood glucose dynamics over the 14-day period are shown in Figure 4. Compared to the C-N group, the C-H group exhibited a significant impairment in early-phase glycemic reduction. Baseline glucose levels (day 0) did not differ between groups. However, blood glucose levels in the C-H group were significantly higher than those in the C-N group at day 4 and day 7 (both *p* < 0.01). This difference was transient. Glucose levels in both groups subsequently converged, showing no significant difference at days 10 and 14. This temporal pattern—early impairment followed by late normalization—preceded the observed downregulation of renal SGLT2 at day 14. Given the multifactorial regulation of fasting glycaemia (e.g., hepatic glucose output, peripheral insulin sensitivity) and the temporal discrepancy (SGLT2 was measured only at the endpoint, whereas glycemic differences emerged as early as day 4), we refrain from attributing a direct causal relationship. Instead, this temporal sequence should be viewed as a hypothesis-generating observation that warrants future functional studies, particularly with direct measurements of 24-h urinary glucose excretion. In addition, the subsequent convergence of fasting blood glucose levels by day 14 suggests the engagement of compensatory homeostatic mechanisms during prolonged hypoxic exposure.

**FIGURE 4:**
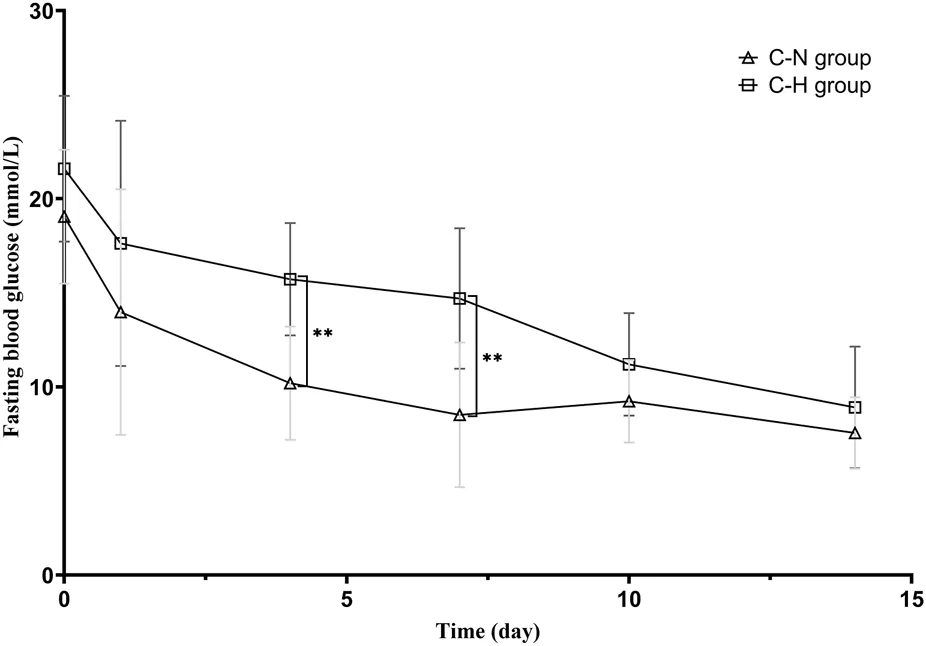
Dynamics of fasting blood glucose levels over time (n = 8). Serial fasting blood glucose levels in chronic normoxia (C–N) and chronic hypoxia (C–H) groups over the 14-day treatment period. T2DM rats received daily dapagliflozin (1 mg/kg/day) throughout. Data are presented as mean ± SD (n = 8 per group). ** Statistical significance was assessed by independent samples t-test at each time point; *p* < 0.01 (C-H vs. C-N). Fasting blood glucose was measured after a 12-h fast from tail vein blood using a portable glucometer.

### 3.4 Effect of chronic hypoxia on renal SGLT2 protein expression

Renal cortical SGLT2 protein expression at the study terminus is summarized in Figure 5. Chronic hypoxia induced a significant downregulation of SGLT2 protein. The mean SGLT2 concentration in the C-H group was 283.30 ± 31.10 ng/L. This value was markedly lower than the 348.48 ± 28.66 ng/L observed in the C-N group (*p* < 0.001).

**FIGURE 5:**
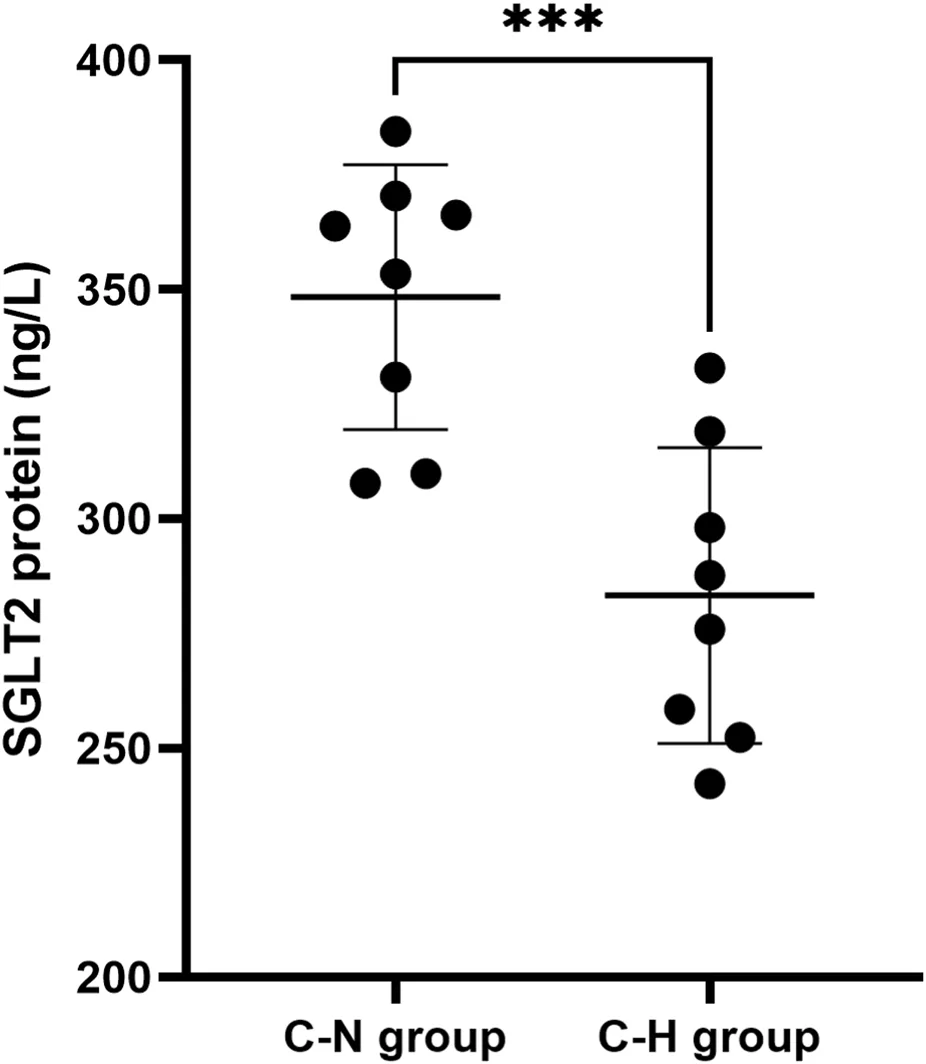
Renal cortical SGLT2 protein expression at study terminus (day 14) in chronic normoxia (C–N) and chronic hypoxia (C–H) groups. SGLT2 concentrations were quantified by ELISA in renal cortex homogenates. Data are presented as mean ± SD (n = 8 per group). ***p < 0.001 (C-H vs. C-N, independent samples t-test).

An apparent directional discordance warrants attention before the Discussion. Pharmacologically, reduced SGLT2 abundance would be expected to enhance glycaemic lowering by diminishing glucose reabsorption. However, we observed lower SGLT2 protein levels coexisting with higher fasting blood glucose during the early treatment phase—a counterintuitive pattern. Given that the glycemic difference emerged on days 4–7 whereas SGLT2 was measured only at day 14, this temporal mismatch precludes a causal interpretation.

### 3.5 Individual-level exposure–response analysis

To examine whether inter-individual variability in dapagliflozin exposure explained the variability in glycemic response, individual AUC_0-t_ and C_max_ were related to fasting blood glucose at the corresponding time point (day 14; Figure 6). When C-N and C-H rats were pooled (n = 16), no significant correlation was observed between AUC_0-t_ and day-14 fasting blood glucose (Spearman ρ = −0.031, *p* = 0.909) or between C_max_ and day-14 fasting blood glucose (Spearman ρ = 0.012, *p* = 0.965). Similar null results were obtained for the increment-adjusted metrics AUC_inc and C_max__inc (all *p* > 0.65; Supplementary Table S1).

**FIGURE 6:**
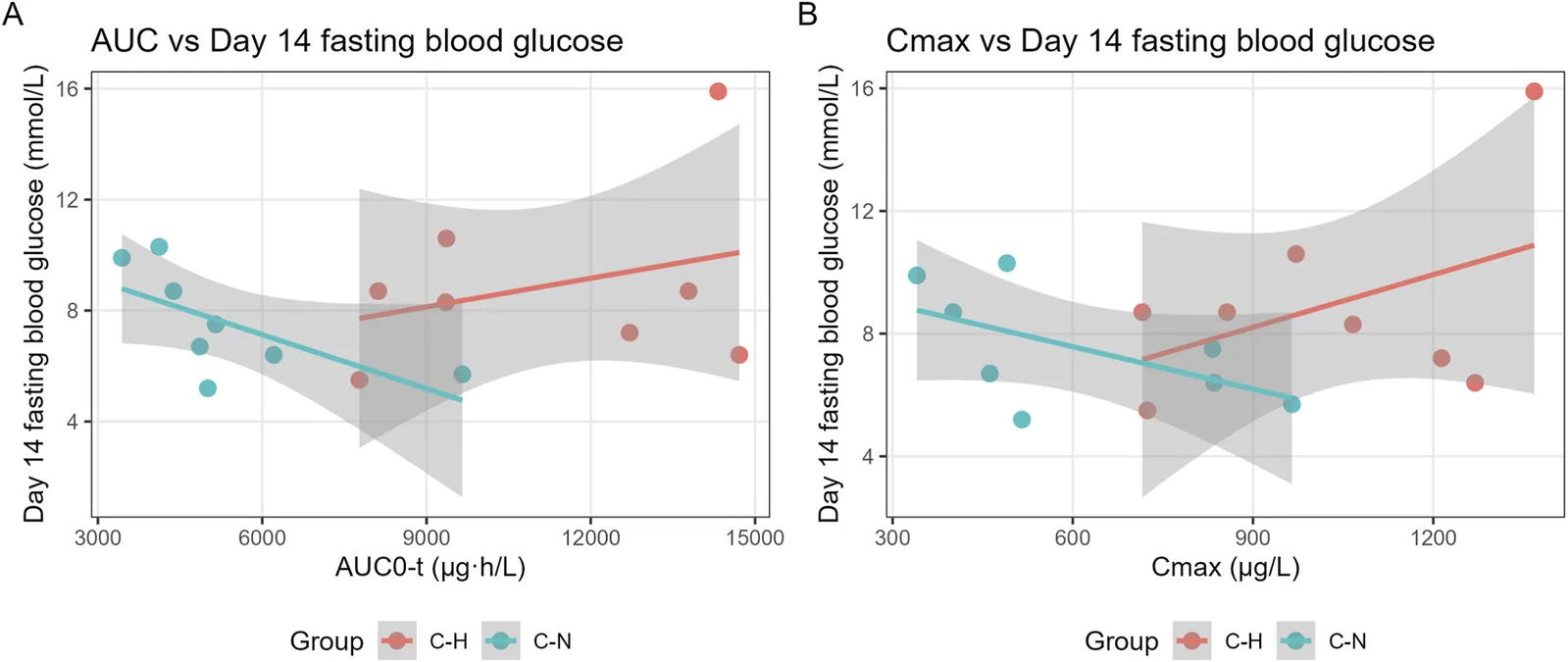
Individual-level exposure–response analysis in chronic normoxia (C–N) and chronic hypoxia (C–H) rats. **(A)** AUC_0-t_ *versus* day-14 fasting blood glucose **(B)** C_max_ *versus* day-14 fasting blood glucose. Each point represents one rat (n = 8). Lines represent linear regression with 95% confidence intervals. Spearman correlation coefficients and *p* values are shown in Supplementary Table S1. PK exposure was measured on day 14, the time point corresponding to the glycemic measurement.

Because pooling across groups may obscure within-group relationships, group-stratified analyses were performed. In the C-N group (n = 8), AUC_0-t_ was significantly negatively correlated with day-14 fasting blood glucose (Spearman ρ = −0.786, *p* = 0.021), and C_max_ showed a borderline negative correlation (Spearman ρ = −0.643, *p* = 0.086), consistent with the expected exposure–response relationship. In contrast, no such relationship was observed in the C-H group (n = 8) (AUC_0-t_: Spearman ρ = 0.240, *p* = 0.568; C_max_: Spearman ρ = 0.144, *p* = 0.734). Linear models including group as a covariate showed no independent association between exposure and day-14 fasting blood glucose after accounting for group (exposure-term *p* = 0.785–0.891; group-term *p* = 0.415–0.627).

Exploratory analyses relating day-14 exposure metrics to day-4 and day-7 fasting blood glucose, as well as to Δfasting blood glucose, showed no significant correlations (all *p* > 0.10; Supplementary Table S1). These analyses should be interpreted cautiously because PK exposure was measured only on day 14 and therefore does not strictly correspond to the earlier glycemic time points. Exploratory day-7 data are shown in Supplementary Figure S1.

## 4 Discussion

This study provides a systematic investigation into the impact of high-altitude hypoxia on dapagliflozin pharmacology. The central finding is a time-dependent pattern of pharmacokinetic (PK) alteration, coupled with significant pharmacodynamic (PD) changes under chronic hypoxia.

### 4.1 Time-dependent pharmacokinetic adaptation: acute insult *versus* chronic attenuation

The present study reveals a clear time-dependent pattern in dapagliflozin pharmacokinetics under high-altitude hypoxia. Acute 3-day exposure provoked a profound PK perturbation, whereas chronic 14-day exposure produced a markedly attenuated effect—a pattern quantitatively evidenced by the ∼70% reduction in fold-change magnitude for systemic exposure (AUC_0-t_) when comparing chronic *versus* acute hypoxia (detailed in Results 3.2). The statistically significant interaction between Hypoxic Treatment and Exposure Duration for AUC_0-t_, Vz/F, and CLz/F further validates that the hypoxic effect on drug disposition is duration-dependent rather than a fixed, sustained perturbation.

Mechanistically, the profound reduction in CLz/F during acute hypoxia may involve acute hemodynamic redistribution (reduced hepatic/renal perfusion) and/or possible alterations in UGT1A9-mediated metabolism, the primary enzyme responsible for dapagliflozin glucuronidation (Zidan et al., 2025; Zhang et al., 2025; Zhang et al., 2020). However, UGT1A9 expression or activity was not directly measured in this study, and this mechanism remains speculative. Because dapagliflozin was administered orally, CLz/F and Vz/F are apparent parameters that cannot distinguish changes in oral bioavailability from true changes in systemic clearance or volume of distribution. The parallel reductions in CLz/F and Vz/F, together with the absence of a marked change in terminal half-life, suggest that increased oral bioavailability may contribute substantially to the observed exposure increase. Therefore, we avoid attributing the effect to suppression of systemic clearance. Conversely, the partial restoration of apparent oral clearance during chronic hypoxia is consistent with known acclimatization processes (Burtscher et al., 2025), including erythropoiesis, cardiac output recovery, and potential HIF-mediated transcriptional reprogramming of metabolizing enzymes (Jaśkiewicz et al., 2023; Midha et al., 2023; Zhao et al., 2023). However, the present study does not provide direct experimental evidence for these molecular pathways; rather, our data establish a descriptive framework of time-dependent PK adaptation that requires dedicated mechanistic investigations.

It is noteworthy that the interaction term reached significance for AUC_0-t_ but not for AUC_0-_ ∞ , a discrepancy likely reflecting greater inter-individual variability in the extrapolated terminal phase. Accordingly, our primary interpretation of time-dependent attenuation relies on AUC_0-t_ and C_max_, which are less influenced by extrapolation (Kasichayanula et al., 2014; Obermeier et al., 2010). The near-complete recovery of CLz/F between hypoxic groups (A-H vs. C-H, *p* = 0.869) is particularly noteworthy, as it points toward recovery of apparent oral clearance rather than mere statistical regression.

The pharmacokinetic parameters observed in the normoxic control groups in the present study are broadly consistent with previously published data on dapagliflozin in normoxic rats (Obermeier et al., 2010). In contrast, both acute and chronic hypoxic groups showed higher systemic exposure than the normoxic controls. Although direct comparison across studies should be made with caution because of differences in dose, sampling schedule, and rat strain, the persistent increase in exposure under chronic hypoxia suggests that repeated dosing does not fully normalize dapagliflozin pharmacokinetics. This observation supports the need for dedicated repeated-dose pharmacokinetic studies under controlled hypoxic conditions.

### 4.2 Pharmacodynamic alterations under chronic hypoxia: glycemic dynamics, SGLT2 downregulation, and PK-PD dissociation

Chronic hypoxia significantly delayed the early-phase reduction of fasting blood glucose (days 4–7), while concurrently downregulating renal cortical SGLT2 protein expression at day 14. However, because the glycemic difference emerged before the SGLT2 measurement time point, this association is correlational rather than causal. The pharmacological scenario can be more usefully disentangled into two distinct axes: renal target modulation and systemic glycemic regulation. The impaired early-phase glycemic reduction observed on days 4–7 may involve contributions from systemic alterations—such as enhanced hepatic gluconeogenesis or peripheral insulin resistance under chronic hypoxia—rather than being a direct consequence of SGLT2 downregulation. We further performed an individual-level exposure–response analysis (Figure 6; Supplementary Table S1). In the C-N group, AUC_0-t_ was significantly negatively correlated with day-14 fasting blood glucose, whereas C_max_ showed a similar but non-significant negative trend, consistent with the expected pharmacodynamic relationship. In contrast, neither AUC_0-t_ nor C_max_ was significantly correlated with day-14 fasting blood glucose in the C-H group. However, because PK exposure was measured only on day 14, we cannot exclude the possibility that differences in dapagliflozin exposure contributed to the early glycemic differences. Therefore, the relative contributions of systemic drug exposure and renal SGLT2 abundance remain difficult to separate, and the early glycemic differences should be interpreted as hypothesis-generating.

These findings reframe the apparent dissociation as a disruption of exposure–response coupling rather than a simple shift in the exposure–response curve, while still allowing for concurrent, independently driven phenotypic changes. Consistent with this view, clinical studies have reported attenuated antihypertensive responses to renin–angiotensin system blockers at high altitude without corresponding changes in drug target expression (Zuo et al., 2026), and losartan failed to lower blood pressure at 5035 m despite its established sea-level efficacy (Lucas et al., 2021). These converging findings suggest that chronic hypoxia fundamentally alters end-organ responsiveness through mechanisms that transcend specific drug classes.

A critical directional discordance warrants attention: lower SGLT2 protein levels at day 14 were associated with higher fasting blood glucose during the early treatment phase (days 4–7), which appears counterintuitive from a purely pharmacological perspective. While the present data do not resolve this paradox, four non-mutually-exclusive explanations could be considered. First, SGLT2 downregulation at day 14 may represent a compensatory response to sustained hyperglycemia or chronic hypoxia, occurring after—rather than causing—the early impairment of glycemic control; this temporal precedence is supported by the fasting blood glucose difference emerging before the protein change. Second, total SGLT2 protein concentration measured by ELISA in whole-tissue homogenates may not reflect its functional activity at the apical membrane; post-translational modifications, membrane trafficking dynamics, or interactions with other transporters such as SGLT1 could offset or even reverse the relationship between total abundance and net glucose reabsorptive capacity (Kasichayanula et al., 2014; Tangri et al., 2024). Third, the net glycemic effect is determined by multiple factors beyond renal glucose handling, including hepatic glucose production and peripheral insulin sensitivity, both of which have been reported to be altered under chronic hypoxia (Woolcott et al., 2015). Fourth, the observed reduction in total SGLT2 protein does not necessarily equate to diminished functional inhibition by dapagliflozin, as the drug’s inhibitory effect depends on target occupancy at the membrane rather than total tissue abundance. Direct measurement of 24-h urinary glucose excretion, SGLT2 membrane localization, and whole-body glucose fluxes will be required to resolve this directional paradox in future investigations.

From a physiological perspective, the observed SGLT2 downregulation under chronic hypoxia may represent a form of renal adaptation rather than merely a pharmacodynamic obstacle. Reduced SGLT2 expression could serve as a homeostatic mechanism to limit excessive glucose reabsorption, thereby conserving glucose for hypoxia-sensitive tissues (e.g., brain, heart) or reducing the osmotic load on kidneys under altered renal hemodynamics (Zhang et al., 2025; Woolcott et al., 2015). This interpretation is supported by the eventual convergence of glycemic levels in the chronic hypoxia group by day 14—suggesting that systemic compensatory mechanisms may progressively offset the initial pharmacodynamic impairment.

### 4.3 Translational implications for high-altitude diabetes management

These preclinical findings, interpreted alongside the human evidence cited above (Zuo et al., 2026; Lucas et al., 2021), suggest two pragmatic considerations for clinical practice.

For individuals with T2DM during rapid ascent (acute exposure): The extreme rise in drug exposure observed in our rat model—6.58-fold increase in AUC—suggests a substantially increased risk of dose-dependent adverse effects. While direct human data for dapagliflozin are lacking, the magnitude of this PK perturbation warrants heightened clinical vigilance. Clinicians should consider enhanced monitoring for patients taking SGLT2 inhibitors during rapid altitude gain, and dedicated clinical studies are needed to determine whether pre-emptive dose adjustment is warranted.

For long-term high-altitude residents with T2DM (chronic exposure): The sustained 2.11-fold increase in drug exposure, coexisting with renal SGLT2 downregulation and delayed early glycemic response, presents a complex scenario. Strikingly, the human evidence from Zuo et al. (Zuo et al., 2026) demonstrates that chronic plateau exposure significantly attenuates antihypertensive efficacy, directly supporting the concept that standard therapeutic regimens may be insufficient for high-altitude populations. By extension, our findings raise the possibility that the standard dapagliflozin dose may provide suboptimal glycemic control in high-altitude residents due to limited target engagement, while the elevated drug exposure nevertheless poses a potential safety risk. This scenario does not mandate blanket dose adjustment based on current evidence; rather, it underscores the urgent need for dedicated clinical PK-PD studies in high-altitude populations to redefine the therapeutic window for SGLT2 inhibitors in this distinct setting.

### 4.4 Limitations and future directions

Several limitations should be acknowledged when interpreting our findings. First, the absence of 24-h urinary glucose excretion data and the single time-point measurement of SGLT2 (only at day 14) preclude a direct causal link between target downregulation and the early glycemic changes, and limit the quantitative assessment of PK–PD relationships. We performed an exploratory individual-level exposure–response analysis using day-14 PK and fasting blood glucose data. A significant within-group association was observed in C-N but not in C-H rats; however, this finding should be interpreted cautiously given the small sample size and exploratory nature of the analysis. However, because PK exposure was measured only on day 14, associations with earlier fasting blood glucose time points remain exploratory, and the relative contributions of systemic exposure and renal SGLT2 abundance to the observed glycemic changes cannot be fully separated. The relationship between dapagliflozin exposure, SGLT2 expression, and glycemic response should be regarded as hypothesis-generating. Second, the acute and chronic studies differed not only in hypoxia duration but also in dosing regimen (single dose vs. multiple daily doses). Although multiple dosing reached steady state by day 14—which is clinically relevant for chronic therapy—this design difference inherently confounds a pure attribution of the observed pharmacokinetic changes solely to hypoxia duration. Consequently, the observed ‘time-dependent attenuation’ should be interpreted as a comparison between single-dose acute exposure and steady-state chronic exposure, rather than a definitive demonstration of acclimatization-driven recovery from an identical initial insult. Future studies employing a single-dose pharmacokinetic assessment after both 3 and 14 days of hypoxia, or utilizing an osmotic pump for continuous delivery, would be required to fully isolate the effect of exposure duration from the confounding influence of dosing schedule. Third, only male rats were used, precluding assessment of sex differences. In addition, no direct data on UGT expression/activity, regional blood flow, oral bioavailability, HIF target gene expression, or SGLT2 membrane localization were obtained; thus, mechanistic interpretations—including the possible contribution of altered UGT1A9-mediated metabolism or hemodynamic changes—remain speculative. Fourth, the sample size (n = 8 per group) was chosen based on previous similar PK studies without formal power analysis, which may limit the detection of subtler pharmacodynamic effects. Finally, findings in chemically induced T2DM rats require confirmation in human studies, particularly given species-specific responses to hypoxia documented in the literature (Burtscher et al., 2025).

Future research should prioritize: (1) integrated PK–PD studies with functional endpoints (24-h urinary glucose excretion, continuous glucose monitoring) in hypoxic models; (2) elucidation of the molecular links between hypoxia, UGT suppression, and SGLT2 regulation; (3) validation of findings in female models and translation to clinical studies in high-altitude residents; and (4) investigation of whether this dual alteration paradigm extends to other SGLT2 inhibitors and drug classes.

## 5 Conclusion

This study demonstrates a time-dependent pattern in the pharmacokinetics of dapagliflozin under high-altitude hypoxia. Acute hypoxia induces severe pharmacokinetic perturbation; chronic hypoxia leads to an attenuated but significant alteration. Furthermore, chronic hypoxia downregulates renal SGLT2 protein expression, and this downregulation was observed in parallel with an attenuated early glycemic response. Individual-level exposure–response analysis showed that AUC_0-t_ was significantly associated with day-14 fasting blood glucose in normoxic rats but not in chronically hypoxic rats; C_max_ showed only a borderline association, providing within-group evidence for a hypoxia-induced dissociation between drug exposure and glycemic response. However, because PK exposure was measured only on day 14, the relationship between SGLT2 downregulation and the early glycemic response remains associative rather than causal.

These findings, interpreted alongside recent human evidence of attenuated antihypertensive efficacy at high altitude, provide a testable hypothesis regarding the complex pharmacology of SGLT2 inhibitors under chronic hypoxia. While our findings do not yet support a specific dose-adjustment recommendation, they strongly underscore the urgent need for dedicated clinical PK–PD studies in high-altitude populations to determine whether and how SGLT2 inhibitor therapy should be optimized in this distinct setting. Nevertheless, because the acute and chronic studies differed in dosing regimen, these findings should be interpreted as a comparative description of drug exposure under acute stress *versus* steady-state chronic therapy.

## Data Availability

The original contributions presented in the study are included in the article/Supplementary Material, further inquiries can be directed to the corresponding author.

## Supplementary material

SUPPLEMENTARY TABLE S1 Exposure-response correlations between dapagliflozin metrics and fasting blood glucose at days 14/4/7 in C-N and C-H groups.

SUPPLEMENTARY FIGURE S1 Day-7 fasting blood glucose vs dapagliflozin exposure in C-N and C-H groups.
