# Supplementary material for: Time-dependent pharmacokinetic changes and renal SGLT2 expression alterations of dapagliflozin under high-altitude hypoxia in diabetic rats
Source: Front Pharmacol. 2026 Oct 26;17:1931102. doi: 10.3389/fphar.2026.1931102 (PMC13745717; doi:10.3389/fphar.2026.1931102)
Supplement: Supplementary file 1 [file Table1.docx]

**Table S1. Individual-level exposure–response analysis of dapagliflozin in chronic normoxia (C-N) and chronic hypoxia (C-H) rats.**

| Analysis | Group | Exposure | Response | n | Spearman ρ | *p* |
| --- | --- | --- | --- | --- | --- | --- |
| Primary | C-N + C-H | AUC_0-t_ | Day 14 fasting blood glucose | 16 | −0.031 | 0.909 |
| Primary | C-N + C-H | C_max_ | Day 14 fasting blood glucose | 16 | 0.012 | 0.965 |
| Primary | C-N + C-H | AUC_inc | Day 14 fasting blood glucose | 16 | −0.121 | 0.655 |
| Primary | C-N + C-H | C_max_ _inc | Day 14 fasting blood glucose | 16 | −0.052 | 0.849 |
| Stratified | C-N | AUC_0-t_ | Day 14 fasting blood glucose | 8 | −0.786 | 0.021 |
| Stratified | C-N | C_max_ | Day 14 fasting blood glucose | 8 | −0.643 | 0.086 |
| Stratified | C-H | AUC_0-t_ | Day 14 fasting blood glucose | 8 | 0.24 | 0.568 |
| Stratified | C-H | C_max_ | Day 14 fasting blood glucose | 8 | 0.144 | 0.734 |
| Exploratory | C-N + C-H | AUC_0-t_ | Day 4 fasting blood glucose | 16 | 0.396 | 0.129 |
| Exploratory | C-N + C-H | C_max_ | Day 4 fasting blood glucose | 16 | 0.267 | 0.318 |
| Exploratory | C-N + C-H | AUC_0-t_ | Day 7 fasting blood glucose | 16 | 0.365 | 0.164 |
| Exploratory | C-N + C-H | C_max_ | Day 7 fasting blood glucose | 16 | 0.2 | 0.457 |
| Exploratory | C-N + C-H | AUC_0-t_ | Δ fasting blood glucose day 4 | 16 | 0.197 | 0.464 |
| Exploratory | C-N + C-H | C_max_ | Δ fasting blood glucose day 4 | 16 | 0.135 | 0.617 |
| Exploratory | C-N + C-H | AUC_0-t_ | Δ fasting blood glucose day 7 | 16 | 0.253 | 0.345 |
| Exploratory | C-N + C-H | C_max_ | Δ fasting blood glucose day 7 | 16 | 0.121 | 0.656 |
| Exploratory | C-N + C-H | AUC_0-t_ | Δ fasting blood glucose day 14 | 16 | −0.021 | 0.94 |
| Exploratory | C-N + C-H | C_max_ | Δ fasting blood glucose day 14 | 16 | −0.001 | 0.996 |

*Note:* Primary analyses examined Spearman rank correlations between day-14 dapagliflozin exposure metrics (AUC_0-t_, C_max_, AUC_inc, and C_max__inc) and day-14 fasting blood glucose in pooled C-N + C-H rats (n = 16). Stratified analyses examined AUC_0-t_ and C_max_ versus day-14 fasting blood glucose separately in C-N and C-H rats (n = 8 per group). Exploratory analyses examined AUC_0-t_ and C_max_ versus day-4 and day-7 fasting blood glucose and versus change from baseline (Δ) in fasting blood glucose at days 4, 7, and 14 in pooled C-N + C-H rats (n = 16). ρ, Spearman correlation coefficient; *p*, two-sided p value without adjustment for multiple comparisons. Pharmacokinetic (PK) exposure was measured only on day 14; therefore, exploratory associations with earlier fasting blood glucose time points should be interpreted cautiously.
